# Identification of TEX101-associated Proteins Through Proteomic Measurement of Human Spermatozoa Homozygous for the Missense Variant rs35033974*[Fn FN2]

**DOI:** 10.1074/mcp.RA118.001170

**Published:** 2018-11-14

**Authors:** Christina Schiza, Dimitrios Korbakis, Keith Jarvi, Eleftherios P. Diamandis, Andrei P. Drabovich

**Affiliations:** From the ‡Department of Laboratory Medicine and Pathobiology, University of Toronto, Toronto, Canada;; §Department of Pathology and Laboratory Medicine,; ¶Lunenfeld-Tanenbaum Research Institute,; ‖Department of Surgery, Division of Urology, Mount Sinai Hospital, Toronto, Canada;; **Department of Clinical Biochemistry, University Health Network, Toronto, Canada

**Keywords:** Proteogenomics, Protein Identification, Label-free Quantification, Clinical Proteomics, Parallel Reaction Monitoring, Selected Reaction Monitoring, Immunoaffinity, rs35033974, Single Nucleotide Variant, Spermatozoa, Testis-specific Proteins, TEX10

## Abstract

TEX101 is a germ-cell-specific protein and a validated biomarker of male infertility. Mouse TEX101 was found essential for male fertility and was suggested to function as a cell surface chaperone involved in maturation of proteins required for sperm migration and sperm–oocyte interaction. However, the precise functional role of human TEX101 is not known and cannot be studied *in vitro* due to the lack of human germ cell lines. Here, we genotyped 386 men for a common missense variant rs35033974 of *TEX101* and identified 52 heterozygous and 4 homozygous men. We then discovered by targeted proteomics that the variant allele rs35033974 was associated with the near-complete degradation (>97%) of the corresponding G99V TEX101 form and suggested that spermatozoa of homozygous men could serve as a knockdown model to study TEX101 function in humans. Differential proteomic profiling with label-free quantification measured 8,046 proteins in spermatozoa of eight men and identified eight cell-surface and nine secreted testis-specific proteins significantly down-regulated in four patients homozygous for rs35033974. Substantially reduced levels of testis-specific cell-surface proteins potentially involved in sperm migration and sperm–oocyte interaction (including LY6K and ADAM29) were confirmed by targeted proteomics and Western blotting assays. Because recent population-scale genomic data revealed homozygous fathers with biological children, rs35033974 is not a monogenic factor of male infertility in humans. However, median TEX101 levels in seminal plasma were found fivefold lower (*p* = 0.0005) in heterozygous than in wild-type men of European ancestry. We conclude that spermatozoa of rs35033974 homozygous men have substantially reduced levels of TEX101 and could be used as a model to elucidate the precise TEX101 function, which will advance biology of human reproduction.

## Abstract (precis)

TEX101 protein is a validated biomarker of male infertility and a potential germ cell-surface chaperone. Near-complete degradation of variant G99V TEX101 protein was discovered in men homozygous and heterozygous for the missense variant rs35033974. Differential proteomic profiling revealed TEX101-associated proteins down-regulated in rs35033974^hh^ spermatozoa, including LY6K protein.

## Graphical Abstract

**Figure:**
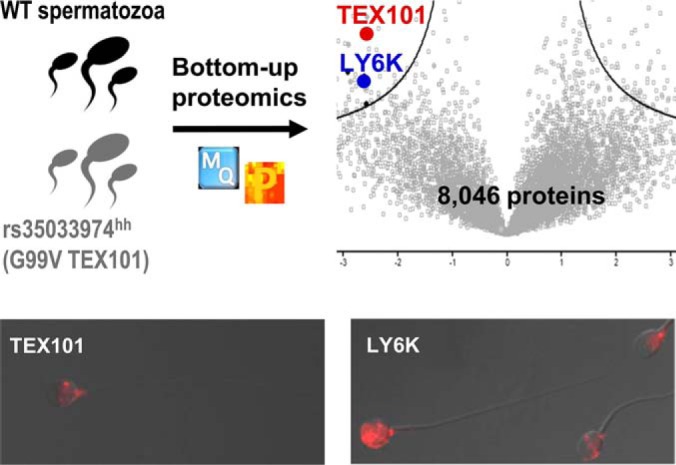

**Highlights**

- Missense variant rs35033974 resulted in significantly reduced levels of human TEX101 protein in seminal plasma and spermatozoa.
- Differential proteomics revealed TEX101-associated testis-specific proteins, including LY6K, which were down-regulated in rs35033974^hh^ spermatozoa.
- Deep proteome of human spermatozoa, including some “missing” proteins, was identified.

Recent -omics studies identified 1,079 human genes with exclusive expression in testis (1). While function of many of testis-specific proteins is not known, it may be assumed that these proteins have unique and specialized roles in spermatogenesis and fertilization. Mutations, natural knockouts, or deleterious single nucleotide variations in testis-specific genes could lead to spermatogenesis arrest, reduced sperm concentration or motility, abnormal sperm morphology, or impaired sperm–oocyte interaction (2–4).

We previously discovered and validated a germ-cell-specific protein TEX101^1^ as a seminal plasma biomarker for the differential diagnosis of azoospermia and male infertility (5–8). The precise functional role of TEX101 is not known, but based on mouse models it was suggested as a testicular germ-cell-surface chaperone involved in the maturation of four cell-surface proteins from the ADAM family (9, 10). *Tex101* knockout in mice resulted in male sterility but normal sperm concentration, morphology, and other phenotypical characteristics (9). In the absence of TEX101 protein, ADAM 3–6 proteins were not properly processed and degraded. However, mouse data could not be translated into human studies because ADAM3, ADAM5, and ADAM6 genes are noncoding pseudogenes, while ADAM4 is not present in the human genome (11). Lack of stable human male germ cell lines hinders identification of TEX101-associated proteins in humans.

As an alternative, we suggested that the functional role of TEX101 could be studied in human clinical samples, such as spermatozoa. Our previous work on TEX101 levels in seminal plasma revealed a small population of men with high sperm count but very low levels of TEX101 protein in seminal plasma and spermatozoa (8). In this work, we hypothesized that some genomic alterations, such as natural knockouts or single nucleotide variations, could result in undetectable or low levels of TEX101 protein. We suggested that spermatozoa obtained from such men could be used as knockout or knockdown models to identify proteins degraded in the absence of TEX101 and discover the functional interactome of TEX101 in humans. Collectively, such data could support in humans the previously suggested function of TEX101 as a cell-surface chaperone (9).

## EXPERIMENTAL PROCEDURES

### 

#### 

##### Study Design and Statistical Rationale

The objectives of this study were to identify potential genomic alterations that could impact levels of TEX101 protein and verify those levels experimentally in human spermatozoa samples. According to power calculations (one-tailed Fisher's exact test, a = 0.05 and 80% power), at least 25 men in each group (prevasectomy and unexplained infertility) would be required to detect an increase of rs35033974^hh^ prevalence from 1.5% (prevalence in the general population) to 28.6% (hypothetical prevalence in men with unexplained infertility). The latter number was calculated as a ratio of rs35033974^hh^ prevalence (1.5%) *versus* the prevalence of unexplained male infertility in the general population (70% of 7.5%) (12). Furthermore, we suggested that differential proteomic profiling of rs35033974^hh^ spermatozoa could identify proteins degraded in the absence of TEX101. According to power calculations, differential profiling of spermatozoa of four wild-type (WT) and four rs35033974 homozygous men could identify proteins down-regulated at least 2.4-fold, assuming 80% power, α = 0.05, 1.8% coefficient of variation for log2-transformed LFQ intensity values, and a one-tailed *t* test (G*Power software, v3.1.7, Heinrich Heine University Dusseldorf). GraphPad Prism (v5.03) was used to generate scatter plots, perform statistical analysis, and calculate receiver operating characteristic area under the curves. Nonparametric Mann–Whitney *U* test was used to compare TEX101 levels in seminal plasma of WT and heterozygous men, and *p* values <0.05 were considered statistically significant.

##### Study Population and Sample Collection

Semen samples (*n* = 386) were collected with informed consent from patients with the approval of the institutional review boards of Mount Sinai Hospital (approval #08–117-E) and University Health Network (#09-0830-AE). Samples were obtained from healthy fertile men before vasectomy and individuals diagnosed with oligospermia or unexplained infertility. Clinical parameters are summarized in Table I. The unexplained infertility group included men who were not able to father a pregnancy after one year of regular unprotected intercourse, with normal sperm concentration of greater than 15 million/ml. After liquefaction, semen samples were centrifuged three times at 13,000 *g* for 15 min at room temperature. Spermatozoa and SP were separated and stored at −80 °C. Samples were analyzed retrospectively. For the differential proteomic analysis, spermatozoa samples were obtained from four men homozygous for *TEX101 c.296G*>*T* variant (rs35033974*^hh^*), diagnosed with oligospermia (*n* = 2) and unexplained infertility (*n* = 2), median age of 29.5 years, sperm concentration 2–30 million/ml, and TEX101 concentration in SP 3.5–47 ng/ml. Spermatozoa obtained from the age-matched WT fertile men referred for vasectomy (*n* = 4, sperm concentration >15 million/ml and TEX101 concentration in SP of 8,000–12,500 ng/ml) were selected as a control group.

##### Extraction of Genomic DNA from Spermatozoa and TEX101 Genotyping

Genomic DNA was extracted from spermatozoa using QIAamp DNA Mini Kit (Qiagen, Inc.). Spermatozoa were washed twice with phosphate-buffered saline (PBS). Cells were lysed in the presence of proteinase K and DNA bound to the membrane was washed and eluted. DNA purity and concentration were measured by spectrophotometer (NanoDrop 8000, Thermo Scientific). Forward (5′-ACAGGACTGAGACAGCCAT-3′) and reverse (5′-TCCAGGGTACCTGTGGTCTC-3′) primers were designed to amplify a 197 base pair fragment of *TEX101* gene encompassing the rs35033974 polymorphism. Polymerase chain reaction (PCR) was performed with 50 ng of genomic DNA, 1.2 units of Phusion High-Fidelity DNA polymerase (Thermo Scientific) in Phusion HF Buffer, 200 μm deoxynucleoside triphosphates, and 0.5 μm primers using mastercycler thermal cycler (Eppendorf). PCR included an initial denaturation step at 98 °C for 1 min, followed by 40 cycles of denaturation at 98 °C for 10 s, annealing at 64 °C for 30 s and extension at 72 °C for 30 s, with a final extension at 72 °C for 7 min. PCR products were confirmed with 1.5% agarose gel electrophoresis and purified with QIAquick PCR Purification Kit (Qiagen). Sequencing of PCR products (*n* = 386 men) was performed by the Centre for Applied Genomic (Hospital for Sick Children, Toronto).

##### Sample Preparation and Protein Digestion by Endopeptidase Glu-C

Spermatozoa pellets were washed twice with PBS, lysed with 0.1% RapiGest SF (Waters, Milford, MA) in 50 mm ammonium bicarbonate, and sonicated three times for 30 s. Cell lysates were then centrifuged at 15,000 *g* for 15 min at 4 °C. Total protein in each spermatozoa or SP sample was measured by the bicinchoninic acid assay. Ten μg of total protein per patient sample in 50 mm ammonium bicarbonate were used for protein digestion. RapiGest SF 0.05% with 5 mm dithiothreitol at 65 °C for 30 min were used to denature proteins (purified recombinant human rhTEX101 and proteins from spermatozoa and SP) and reduce disulfide bonds. Free thiols were then alkylated with 10 mm iodoacetamide in the dark for 40 min at room temperature. Protein digestion was completed overnight at 37 °C in the presence of sequencing grade Glu-C enzyme obtained from Promega (1:20 Glu-C: total protein) and supplemented with 5% acetonitrile to enhance Glu-C activity. Digestion in the presence of ammonium bicarbonate at pH 7.8 ensured specific cleavage after glutamine residues. Trifluoroacetic acid (1%) was then used to inactivate Glu-C and cleave RapiGest SF detergent. Synthetic peptides representing the WT (AITIVQHSSPPGLIV*TSYSNYCE) and the G99V variant (AITIVQHSSPPVLIV*TSYSNYCE) forms of TEX101 were labeled with 13C5-, 15N-valine at the residue 102 and were used as internal standards spiked-in after digestion at final concentrations of 100 fmol/μ*l and 250 fmol*/μ*l, respectively. Digests were desalted, and peptides were extracted by C18 OMIX tips (Varian, Inc., Lake Forest, CA). Peptides were eluted into 3* μ*l of 70*% *acetonitrile with 0.1*% *formic acid and analyzed by an EASY-nLC 1000 nanoLC coupled to Q Exactive*^TM^ Plus Hybrid Quadrupole-Orbitrap^TM^ Mass Spectrometer (Thermo Fischer Scientific).

##### Development of Parallel Reaction Monitoring (PRM) Assay for WT and G99V Variant forms of TEX101 Protein

To evaluate Glu-C specificity and efficiency of digestion, rhTEX101 protein was digested and analyzed in the data-dependent discovery mode. Raw files were analyzed using the Proteome Discoverer™ software (Thermo Scientific, version 1.4.1.14), and specific generation of AITIVQHSSPPGLIVTSYSNYCE peptide (*m/z* = 1,268.6) was confirmed. Uniqueness of these peptides in the human proteome was confirmed by Basic Local Alignment Search Tool (http:/blast.ncbi.nlm.nih.gov/Blast.cgi). Following that, rhTEX101 and SP were digested with Glu-C and analyzed in the unscheduled targeted PRM mode. In the final optimized PRM method, heavy-isotope-labeled peptide internal standards and an additional endogenous TEX101 peptide TAILATKGCIPE (*m/z* = 637.3) were monitored (supplemental Table S1). A four-step 16-min gradient was used: 20% to 40% of buffer B for 8 min, 40% to 65% for 2 min, 65% to 100% for 2 min, and 100% for 4 min. PRM settings were the following: 3.0 eV in-source collision-induced dissociation (CID), 17,500 MS2 resolving power at 200 *m/z*, 3 × 10^6^ automatic gain control (AGC) target, 100 ms injection time, 2.0 *m/z* isolation window, optimized collision energy at 27, and 100 ms scan times.

##### Immunocapture-PRM Measurements of WT and G99V TEX101

Total TEX101 protein was enriched from SP and spermatozoa using an in-house anti-TEX101 mouse monoclonal antibody 34ED556. Briefly, protein G purified 34ED556 monoclonal antibody was immobilized on N-hydroxysuccinimide (NHS)-activated Sepharose 4 Fast Flow beads (GE Healthcare). Fifty μl of beads (∼25 μg of 34ED556) in 0.1% BSA were incubated overnight at 4 °C with seminal plasma or spermatozoa lysate. After binding, beads were washed three times with tris buffer saline (50 mm Tris, 150 mm NaCl, pH 7.5) followed by washing with 50 mm ammonium bicarbonate. Proteins were digested overnight on beads using Glu-C. Supernatants were acidified with 1% TFA. Heavy peptides (200 fmol of WT and 500 fmol of G99V) were spiked into each sample after digestion. Digests were desalted, and peptides were measured by PRM assay. Raw files were analyzed with Skyline software (v3.6.0.10493), and the relative abundances of WT or G99V variant TEX101 forms were calculated using the light-to-heavy peptide ratios.

##### Sample Preparation for the Differential Proteomic Analysis

Spermatozoa pellets from eight men (four WT and four rs35033974*^hh^*) were lysed with 0.1% RapiGest SF in 50 mm ammonium bicarbonate. Cell lysates were centrifuged at 15,000 *g* for 15 min at 4 °C to remove debris, and total protein concentration was measured using BCA assay. Proteins (225 μg per sample) were denatured, reduced with 5 mm dithiothreitol, alkylated with 10 mm iodoacetamide, and digested overnight with trypsin (Sigma-Aldrich) at 37 °C.

##### Strong Cation Exchange Chromatography Fractionation

Off-line strong cation exchange chromatography fractionation was used to facilitate deep proteome analysis. Tryptic peptides were diluted with mobile phase A (0.26 m formic acid in 10% acetonitrile [ACN] at pH 2–3) and were loaded onto PolySULFOETHYL A™ column (2.1 mm inner diameter × 200 mm, 5 μm, 200 Å, The Nest Group, Inc., MA). Peptides were separated with a 60-min three-step HPLC gradient (Agilent 1100) and eluted at 200 μl/min with 1 m ammonium formate (0–15% for 5–25 min, 25% at 35 min, and 100% at 50 min). Twenty-seven 400 μl fractions were initially collected but then pooled into 13 fractions based on absorbance profiles.

##### Protein Identification by Liquid Chromatography–tandem Mass Spectrometry (LC-MS/MS)

Peptides of each strong cation exchange chromatography fraction were concentrated with C18 OMIX tips and analyzed by an EASY-nLC 1000 system coupled to a Q Exactive^TM^ Plus mass spectrometer in technical duplicates for each fraction (13, 14). Peptides were separated with a 15-cm C18 analytical column using a 90-min LC gradient at 300 nl/min flow rate. Full MS1 scans (400 to 1,500 *m/z*) were acquired with the Orbitrap analyzer at 70,000 full width at half maximum resolution in the data-dependent mode, followed by 12 data-dependent MS2 scans at 17,500 full width at half maximum. Only +2 and +3 charge states were subjected to MS2 fragmentation.

##### Data Analysis and Label-free Quantification

XCalibur software (v. 2.0.6; Thermo Fisher Scientific) was utilized to generate raw files. For protein identification and label-free quantification, raw files were analyzed with MaxQuant software (version 1.5.2.8). MaxQuant searches were performed against the nonredundant Human UniprotKB/Swiss-Prot database (HUMAN5640_sProt-072016) at 1.0% FDR. Search parameters included: trypsin enzyme specificity, two missed cleavages, minimum peptide length of seven amino acids, minimum identification of one razor peptide, fixed modification of cysteines by carbamidomethylation, and variable modification of methionine oxidation and N-terminal protein acetylation. The mass tolerance was set to 20 ppm for precursor ions and 0.5 Da for fragment ions with top 12 MS/MS peaks per 100 Da. MaxLFQ algorithm facilitated label-free relative quantification of proteins (15). ProteinGroups.txt file was uploaded to Perseus software (version 1.5.5.3) to facilitate statistical analysis (16). Proteins classified as “only identified by site,” “reverse,” and “contaminants” were filtered out, and LFQ intensities were log2-transformed. Missing LFQ values were imputed with the down shift of 1.8 and distribution width of 0.45 to ensure normal distribution, and average LFQ intensities for two technical replicates were calculated. A two-sample *t* test with Benjamini–Hochberg FDR-adjusted *p* values was applied, and 5.0% FDR with calculated constant for variance correction s0 = 0.4 were used to select proteins differentially expressed in rs35033974^hh^ men. Data were visualized with volcano plots. Significant up- or down-regulated proteins were filtered for the cell-surface and secreted proteins with the testicular-tissue-elevated (tissue-enriched, group enriched, and tissue-enhanced) expression according to the Human Protein Atlas, version 13 (1).

##### Experimental Design and Rationale for Development of Selected Reaction Monitoring (SRM) Assays and Quantification of Candidate Proteins

To quantify candidate proteins, we developed and applied Tier 2 SRM assays, as previously described (17–21). Briefly, LC-MS/MS peptide identification data were used to select proteotypic tryptic peptides and develop SRM assays. Choice of peptides was confirmed with the SRM Atlas (www.srmatlas.org). For each protein, peptides with 7–20 amino acids and without missed cleavages were chosen, and heavy-isotope-labeled peptide internal standards were synthesized. Several unscheduled 30-min SRM methods were prepared and run with a pool of spermatozoa digest with TSQ Quantiva^TM^ triple quadrupole mass spectrometer (Thermo Scientific). The three most intense transitions were selected for each heavy or light peptide. Finally, 20 heavy and light peptides were scheduled within 2-min intervals during a 30-min gradient in a single multiplex SRM assay (supplemental Table S2). The parameters for SRM assay included: positive polarity, 150 V declustering and 10 V entrance potentials, 300^°^C ion transfer tube temperature, optimized collision energy values, 20 ms scan time, 0.4 Q1 and 0.7 Q3 full width at half maximum resolutions, and 1.5 mTorr Q2 argon pressure. Because one rs35033974 homozygote spermatozoa sample was fully consumed in the discovery experiment, candidate proteins were quantified in three rs35033974 homozygote and four WT spermatozoa samples. Spermatozoa lysates (10 μg protein) were digested by trypsin. TEX101 and DPEP3 internal standards with trypsin-cleavable tags (500 fmol of AGTETAILATK*-JPTtag and SWSEEELQGVLR*-JPTtag, respectively) were added before trypsin digestion, while eight heavy-isotope-labeled peptides without JPT tags were spiked after digestion (500 fmol each). Stable-isotope-labeled peptides with or without JPTtag (serine-alanine-[3-nitro]tyrosine-glycine) were obtained from JPT Peptide Technologies GmbH (Berlin, Germany). Light and heavy peptides were monitored with a scheduled 30-min multiplex SRM assay. Each spermatozoa sample was analyzed. Light-to-heavy ratio was used to calculate the accurate relative abundance of each candidate protein.

##### Western Blotting

Protein levels of TEX101, LY6K, ADAM29, and DPEP3 were assessed by Western blot analysis. Twenty μg of total protein from one rs35033974 homozygous and one WT spermatozoa lysate were loaded onto an SDS-PAGE gel (4–15%, Bio-Rad) and transferred onto PVDF membranes (Bio-Rad). After blocking, membranes were incubated overnight at 4 °C with rabbit polyclonal antibodies against TEX101 and DPEP3 (HPA041915 and HPA058607, Sigma-Aldrich, St. Louis, MO), sheep polyclonal antibody against LY6K (AF6648, R&D Systems, Minneapolis, MN), mouse monoclonal antibody against ADAM29 (H00011086-M09, Abnova Corporation, Walnut, CA), and GAPDH antibody (AM4300, Thermo Fisher Scientific, Rockford, IL). The membranes were washed then incubated with goat anti-rabbit, donkey anti-sheep, and goat anti-mouse secondary antibody conjugated to horseradish peroxidase (Jackson ImmunoResearch, West Grove, PA). Proteins were detected with chemiluminescence substrate (GE Healthcare Life Sciences, Mississauga, ON).

##### Immunofluorescence Analysis

Immunofluorescence images were taken with an Olympus BX61-Fluo upright fluorescence motorized microscope equipped with a digital camera (Hamamatsu C8484-03G01), a 100× oil immersion super apochromat objective (Olympus UPlanSApo 100XO) and fluorescence filters (DAPI-5060B-OMF and TXRED-4040B-OM, Semrock, Rochester, NY) or with Invitrogen EVOS FL Auto 2 Imaging System equipped with a 60× oil immersion apochromat objective (Olympus PlanApo N 60XO) and fluorescence filters (DAPI and TxRed EVOS light-emitting diode [LED] light cubes). Images were examined and captured using HCImage (v 4.4.5, Hamamatsu, Japan) or EVOS™ FL Auto 2 (Invitrogen, Thermo Fisher Scientific). Exactly the same microscope and software settings were used for positive staining and negative controls. Spermatozoa were immobilized onto glass slides with a cytology fixative (Adwin Scientific, US), dried, blocked with goat serum, and washed with PBS. In-house mouse monoclonal anti-TEX101 antibody 34ED229 (25 μg/ml) or rabbit polyclonal anti-LY6K antibody (7 μg/ml; PAB21148, Abnova, Taiwan) were used as primary antibodies and incubated for 2 h at room temperature. Goat-anti-mouse IgG1 (2 μg/ml final; A-21125; Invitrogen) and goat-anti-rabbit IgG H+L (1 μg/ml; A-11037, Invitrogen) labeled with Alexa Fluor 594 were used as secondary antibodies and were incubated for 1 h at room temperature in the dark. Mountant solution with DAPI (S36973, Invitrogen) was applied overnight.

## RESULTS

### 

#### 

##### Database Mining for Loss-of-function Variants of TEX101 Gene

**Fig. 1.**
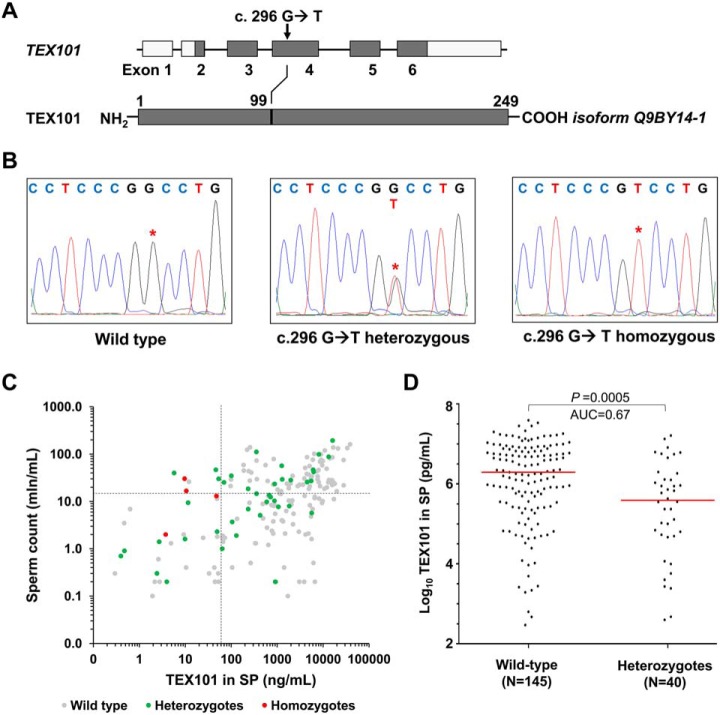
**Identification of *rs35033974* variant in *TEX101* gene.** (*A*) Schematic representation of *TEX101* gene and protein sequence, showing the missense variant c.296 G>T, and the amino acid substitution p.99 G>V. (*B*) *TEX101* DNA sequencing of WT *TEX101* patient (*left panel*), rs35033974 heterozygous patient (*middle panel*) and rs35033974*^hh^* homozygous patient (*right panel*). (*C*) Scatter plot of sperm concentration and TEX101 levels in seminal plasma of 189 subfertile men (diagnosed with unexplained infertility or oligospermia) of European origin. WT, rs35033974 heterozygous and rs35033974*^hh^* men are plotted in gray, green, and red, respectively. Sperm concentration (15 million/ml) and TEX101 (65 ng/ml) cut-off values are indicated by dotted lines in black. Variant rs35033974 allele frequency is higher for the groups with TEX101 < 65 ng/ml (58.3% for upper left group and 21% for lower left group), and lower for the groups with TEX101 ≥ 65 ng/ml (9.8% for lower right group and 8.7% for upper right group). (*D*) Seminal plasma levels of TEX101 normalized by sperm concentration were significantly lower (fold change 4.0, MWU *p* = 0.0005) for G99V heterozygous European men (median 390 ng/ml, *n* = 40), as compared with WT men (median 1,949 ng/ml, *n* = 145). Horizontal lines represent median values for each group.

The Exome Aggregation Consortium (http://exac.broadinstitute.org), Genome Aggregation (http://gnomad.broadinstitute.org), and 1000 Genomes Project (www.internationalgenome.org) databases (22, 23) were examined for the presence of potential loss-of-function variants of the human *TEX101* gene. GnomAD database included genomic variants identified in 138,632 individuals of diverse ethnic background and revealed 166 potential loss-of-function variants of *TEX101* (supplemental Table S3). Protein knockout or truncating variants, such as start loss, stop-gain and frameshift variants, were very rare (minor allele frequencies <0.003%), while some missense variants leading to single amino acid substitutions were much more frequent. One such missense variants was rs35033974 (allele frequency 8.4%). Interestingly, rs35033974 was predicted as “deleterious” by Polyphen (24), sorting intolerant from tolerant (SIFT) (25), and combined annotation dependent depletion (CADD) (26) algorithms. The allele frequency of rs35033974 varied among different populations. It was more common in European non-Finnish population (12.4%), less common in Latino (5.2%), Ashkenazi Jewish (5.8%), South Asian (3.3%), and African (2%), and very rare in the East Asian population (<0.00001%). Rs35033974 (*c. 296 G*>*T*) was localized within exon 4 and resulted in substitution of glycine to valine at position 99 (Fig. 1*A*). Alignment of TEX101 protein sequences suggested that glycine-99 was a conserved residue in 17 of 19 mammals and thus could be intolerant to substitutions (supplemental Fig. S1). The high genotype frequency of rs35033974 (22% hetero- and 1.6% homozygosity in European population) warranted its identification in our spermatozoa biobank.

##### Identification of Men Heterozygous and Homozygous for rs35033974 Variant

Genotypes for rs35033974 variant (c. 296 G>T) were determined in 386 men by amplification of spermatozoa DNA and sequencing analysis (Table I and Fig. 1*B*). Four heterozygous individuals (GT, genotype frequency 11%) were identified in the group of pre-vasectomy fertile men (*n* = 37). In the group of patients with unexplained male infertility (*n* = 175), 23 heterozygous (13%) and 2 (1.1%) homozygous patients (TT) were found. In the group of patients diagnosed with oligospermia (*n* = 174), we identified 25 heterozygous (14%) and 2 homozygous (1.1%) patients. Investigation of patients with European ancestry (178 WT, 44 heterozygous, and 4 homozygous men) revealed minor allele frequency of 11.5% and was similar to gnomAD frequency of 12.4%. In our cohort of European men, the minor allele frequency was not significantly different for fertile men prevasectomy *versus* patients with unexplained infertility and oligospermia (Fisher's exact test *p* = 0.15).

**Table I:** Clinicopathological variables of 386 patients

Clinical parameters	*N*	%
Number of patients	386	100
Median age [range]	41 [19–63]	
Ethnic background of patients		
African-Canadian	15	3.9
Asian	37	9.6
European	226	58.5
Hispanic	6	1.5
Indo-Canadian	6	1.5
Middle Eastern	14	3.6
Native Canadian	5	1.3
Unspecified/unavailable	77	20.1
Diagnosis [range of sperm concentration, mln/ml]		
Fertile pre-vasectomy	37	9.6
Unexplained infertility (15–36 mln/ml)	175 [15–36]	45.3
Oligospermia	174 [0.1–15]	45.1

According to the 1000 Genomes Project, rs35033974 variant was present with the similar allele frequencies in males and females. Interestingly, 1000 Genomes Project data (23) included nine homozygous men, of which five had biological children (see examples in supplemental Fig. S2). Using gnomAD data, we found no substantial deviation from the Hardy–Weinberg equilibrium for homozygous men in the European population (*n* = 63,332; 1,011 homozygotes identified *versus* 982 calculated). Based on 1000 Genomes Project and gnomAD data, as well as minor allele frequencies in our clinical cohorts, we suggested that rs35033974^hh^ is unlikely a monogenic factor of male infertility.

##### Impact of rs35033974 on TEX101 Protein Concentration in Seminal Plasma

We previously measured by ELISA concentration of total TEX101 protein in seminal plasma of 805 men (8). Cross-checking revealed that concentration of total TEX101 in seminal plasma of four rs35033974*^hh^* men was extremely low (3.5 to 47 ng/ml), despite of their medium-to-high sperm concentration (2 to 30 mln/ml; Fig. 1*C*). Here, we also examined possible associations between rs35033974 heterozygous status and TEX101 levels in seminal plasma or sperm concentration in semen. No significant difference for sperm concentration was found for WT *versus* heterozygous European population (MWU *p* value = 0.94). However, levels of TEX101 in seminal plasma were significantly lower for rs35033974 heterozygous men (median 390 ng/ml, MWU *p* = 0.0005, *n* = 40), as compared with WT men of European population (median 1,949 ng/ml, *n* = 145; Fig. 1*D*). Because TEX101 concentration in seminal plasma may correlate with the number of spermatozoa in semen, we also investigated normalized TEX101 concentration. Thus, seminal plasma levels of TEX101 normalized by sperm concentration were significantly lower (fourfold change, MWU *p* < 0.0001) for heterozygous men (median 40,738 attograms/cell, *n* = 40), as compared with WT men (median 164,085 attograms/cell, *n* = 145).

Because TEX101 levels were found significantly lower in heterozygous and homozygous men, rs35033974 status may be considered in the clinical use of TEX101 protein as a biomarker of male infertility (to evaluate vasectomy success, differentiate between nonobstructive and obstructive azoospermia, and predict the success of sperm retrieval in patients with non-obstructive azoospermia) (8). Otherwise, some rs35033974-positive men (for example, 23% men of European population) could be misclassified because their TEX101 levels will be below the established clinical cut-offs (8). In addition, patients with different ethnic backgrounds may have different cut-off values for TEX101 due to differences in rs35033974 frequencies.

##### Rs35033974 Results in Degradation of G99V TEX101 Protein

To monitor the WT and G99V variant forms of TEX101 protein in spermatozoa lysate and SP, we opted to develop a targeted mass spectrometry assay (27, 28). Because TEX101 digestion by trypsin generated a 38-amino-acid peptide not suitable for bottom-up proteomic measurements, we explored alternative proteases, such as endopeptidase Glu-C (cleaves after aspartic and glutamic acids) and neutrophil elastase (cleaves after valine and alanine). Endopeptidase Glu-C was found the most suitable enzyme. Following optimization of Glu-C digestion protocol, we developed a PRM assay to monitor WT and G99V TEX101 peptides, as well as an additional endogenous “control” peptide that represented total TEX101. Sensitivity of PRM assay, however, was not sufficient to measure low levels of TEX101 in seminal plasma (<1.5 μg/ml).

**Fig. 2.**
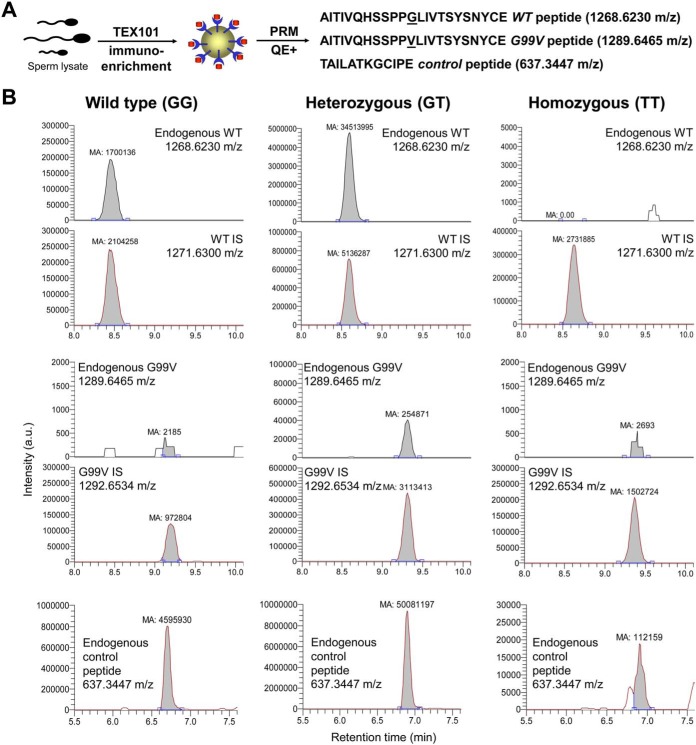
**Measurement of WT and G99V variant forms of TEX101 protein by targeted mass spectrometry.** (*A*) Immuno-PRM assay included immunoenrichment of endogenous TEX101 with a mouse monoclonal antibody 34ED556 coupled to Sepharose beads, followed by Glu-C digestion and PRM measurements of WT and G99V peptides, as well as a control peptide representing total TEX101. (*B*) In a WT patient (GG, *left panel*), high levels of TEX101 were measured in seminal plasma by PRM assay without immunoenrichment. In rs35033974 heterozygote (GT, *middle panel*) and rs35033974*^hh^* homozygote (TT, *right panel*) men, WT, G99V variant, and control peptides of TEX101 were measured by immuno-PRM assay in spermatozoa lysates. Corresponding heavy-isotope-labeled peptides were used as internal standards to ensure correct identification and accurate relative quantification of peaks. Assuming theoretically equal expression of both alleles and similar ionization efficiencies of WT and G99V peptides, <3% of expected G99V variant form was found in spermatozoa of a heterozygous patient, suggesting that G99V TEX101 form may be degraded during spermatogenesis.

To improve assay sensitivity, we developed an immuno-PRM assay based on the immunoenrichment of TEX101 by our in-house anti-TEX101 mouse monoclonal antibody 34ED556 coupled to Sepharose beads (Fig. 2*A*). Using immuno-PRM assay, we were able to identify barely detectable levels of G99V variant protein in one homozygous spermatozoa sample, while the other two homozygous spermatozoa samples had undetectable levels of G99V TEX101. We also selected one heterozygous spermatozoa sample with a very high concentration of TEX101 in seminal plasma (16.1 μg/ml) and were able to measure levels of both WT and G99V variant forms (Fig. 2*B*). Interestingly, the abundance of a G99V variant form was substantially lower, as compared with the WT form. Using heavy-to-light ratios of both peptides, we estimated that the abundance of the G99V form was ∼97% lower than predicted, assuming equal expression of both alleles.

To explain this phenomenon, we suggested that the G99V variant form of TEX101 protein could be misfolded, aggregated, and destroyed through proteasomal degradation (29–31). Similar impact was previously observed for misfolded cystic fibrosis transmembrane conductance regulator (CFTR) (32) and some GPI-anchored proteins (33). A large residue of valine at position 99 could introduce substantial steric constraints, eliminate the PPGL beta-turn, and destabilize beta-sheets in the proximity of G99V (supplemental Fig. S3). Interestingly, the TANGO algorithm (34) revealed a significant impact of G99V substitution on the values of cross-beta aggregation in unfolded proteins (increase from 3.6 to 83.8). The impact predicted by TANGO was the most deleterious for substitutions with hydrophobic residues of valine, isoleucine, and phenylalanine.

##### Global Proteomic Profiling Revealed Testis-specific Proteins Down-regulated in rs35033974^hh^ Spermatozoa

Spermatozoa obtained from four rs35033974*^hh^* men were considered as a *TEX101* functional knockdown model. Taking into account degradation of ADAM3 proteins in spermatozoa of *Tex101* knockout mice (9), we hypothesized that TEX101-associated proteins would be degraded in rs35033974*^hh^* spermatozoa and could be identified by differential proteomic profiling. Unlike immunoprecipitation approaches to identify only direct and strong physical interactions, differential profiling of the whole proteomes of WT *versus* rs35033974*^hh^* spermatozoa could identify strong, weak, transient, and indirect interactions impacted in the absence of TEX101.

**Fig. 3.**
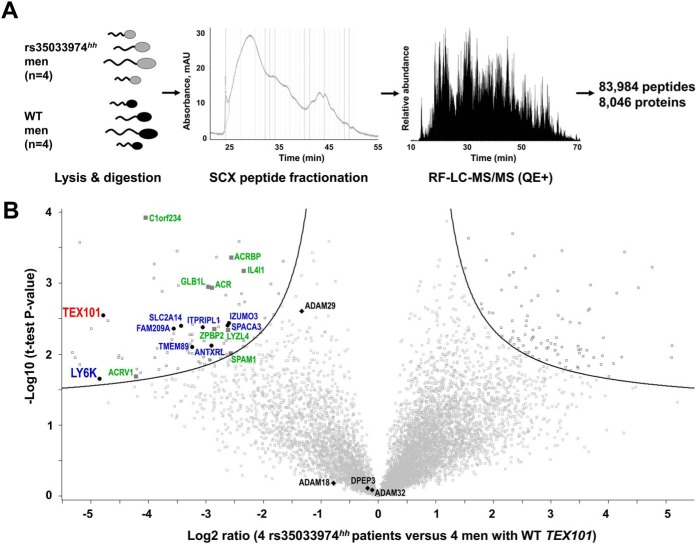
**Identification of proteins down-regulated in rs35033974*^hh^* spermatozoa, as compared with WT spermatozoa.** (*A*) Spermatozoa obtained from four WT and four rs35033974*^hh^* men were digested by trypsin, fractionated by strong-cation exchange chromatography, and analyzed by LC-MS/MS. (*B*) A total of 8,046 protein groups representing 8,473 unique gene names and 8,431 UniProt protein IDs were identified and quantified. Volcano plot revealed proteins down-regulated in rs35033974*^hh^* spermatozoa with FDR ≤5% (hyperbolic curve) calculated using log2-transformed fold change ratios, BH-adjusted *t* test *p* values and s0 = 0.4 variance correction. Differentially expressed testis-specific cell-surface and secreted proteins were plotted in blue and green, respectively. Three proteins with adhesion activity of the ADAM family (ADAM18, ADAM29, and ADAM32) were identified. Levels of DPEP3 (a testis-specific and TEX101-interacting protein not affected by *Tex101* knockout in mice) were not changed. No testis-specific cell-surface or secreted proteins were found significantly up-regulated.

We thus performed a global proteomic analysis of four WT and four rs35033974*^hh^* spermatozoa (Fig. 3*A*). To achieve deep proteome coverage, peptides were subjected to the offline fractionation by strong cation exchange chromatography followed by the online reversed-phase liquid chromatography–mass spectrometry detection. As a result, MaxQuant analysis identified and quantified 83,984 unique peptides and 8,046 protein groups with FDR ≤ 1.0% (supplemental Table S4). Of 189 differentially regulated proteins (FDR ≤ 5.0% and s0 = 0.4), 96 were down-regulated and 93 were up-regulated (Fig. 3*B* and supplemental Table S5). Filtering of these candidates for testis specificity using Human Protein Atlas data revealed 55 down-regulated but only 4 up-regulated proteins (potential false-positive candidates). Thus, many more testis-specific proteins were affected by TEX101 loss. Additional filtering for cell-surface and secreted proteins using NextProt database revealed eight down-regulated cell-surface and nine secreted proteins but zero up-regulated proteins (Fig. 3*B*).

Here, we also hypothesized that differential proteomic analysis of rs35033974*^hh^* spermatozoa could reveal functional orthologs of mouse ADAMs 3–6 proteins degraded in *Tex101* knockout mice (9, 10). In our spermatozoa proteome, we identified seven human testis-specific ADAM proteins, of which three proteins with the adhesion activity (ADAM18, ADAM29, and ADAM32) could be potential orthologs of mouse ADAM 3–6 proteins (35). It was only ADAM29 protein which levels were lower in rs35033974*^hh^* spermatozoa (Fig. 3*B*). Even though ADAM29 did not pass our cut-off criteria of the global differential analysis, it was down-regulated 2.5-fold (Benjamini-Hochberg [BH]-adjusted *t* test *p* = 0.003).

In this work, we identified and quantified one of the largest proteomes of human spermatozoa (8,046 protein groups representing 8,473 unique gene names and 8,431 UniProt protein identifications [IDs]). Of these, 7,156 protein groups and 7,573 unique Uniprot IDs were quantified with two or more unique peptides. Interestingly, of 2,186 proteins currently defined as “missing” by NextProt (v2.14.0), we identified 127 proteins with two unique peptides (101 with previous evidence only at the transcript level, 21 inferred from homology, and 5 predicted). For example, we discovered in our dataset seven testis-elevated missing proteins (ANKRD60, C12orf42, LRRC63, CCDC74B, FAM47C, SPATA31A1, and TTLL8) that were identified with two unique peptides (≥ 9 amino acids) and could represent true proteins according to NextProt criteria. To conclude, our spermatozoa proteome could be used as a resource to identify and update currently missing testis-expressed proteins.

##### Evaluation of Candidates by SRM and Western Blotting

**Fig. 4.**
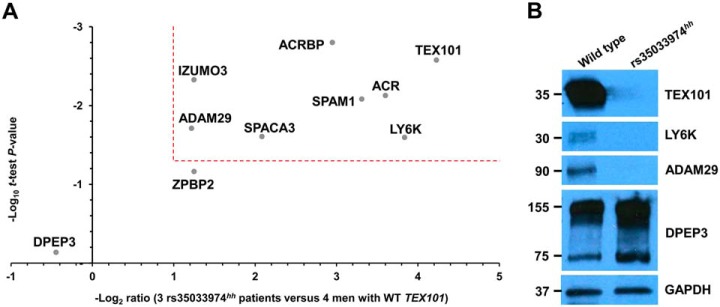
**Verification of 8 testis-specific cell-surface and secreted proteins in four WT and three rs35033974*^hh^* men by SRM and Western blotting.** (*A*) A multiplex SRM assay facilitated accurate relative quantification and confirmed significant down-regulation (>2-fold change, *p* value <0.05, dotted lines) of seven proteins. (*B*) Western blot analysis of spermatozoa obtained from one WT and one rs35033974*^hh^* men confirmed reduced levels of TEX101, LY6K, and ADAM29 proteins, while levels of a testis-specific protein DPEP3, present in spermatozoa as both a monomer and a homodimer, were not affected. GAPDH was used as a loading control for total protein.

Four cell-surface and four secreted testis-specific proteins involved in sperm migration (36), zona pellucida binding and penetration (37–39), and sperm-oocyte fusion (40, 41) were selected for SRM analysis (Table II). Each sample was analyzed in technical duplicates, and the mean light-to-heavy ratios were calculated. The median technical coefficient of variation (CV) values for all patients ranged from 1 to 31% (supplemental Table S6). As a result of SRM measurements, seven of eight proteins were down-regulated in rs35033974^hh^ spermatozoa, while levels of DPEP3 (a testis-specific TEX101-interacting protein not affected by Tex101 knockout in mice (42)), were not changed (Fig. 3*B*, Fig. 4*A*, and Table II). A single-peptide SRM assay revealed an 18-fold decrease of TEX101 protein abundance in rs35033974^hh^ spermatozoa. Such decrease correlated with the 28-fold decrease measured by the global proteomic analysis based on six unique peptides (supplemental Fig. S4). Substantial decrease of TEX101 protein abundance in rs35033974*^hh^* spermatozoa was also confirmed by immunofluorescence analysis (supplemental Fig. S5).

**Table II:** List of down-regulated testis-expressed proteins in spermatozoa of rs35033974*^hh^* homozygous men, as discovered by shotgun mass spectrometry and measured by SRM

UniProt accession	Gene name	Protein	Shotgun log2- fold change	SRM log2- fold change	Protein class
Q9BY14	TEX101	Testis-expressed protein 101 (isoform 1)	–4.8	–4.2	Cell-surface
Q17RY6	LY6K	Lymphocyte antigen 6K (isoform 1)	–4.9	–3.8	Cell-surface
P10323	ACR	Acrosin	–2.9	–3.6	Secreted
Q6X784	ZPBP2	Zona pellucida-binding protein 2	–2.9	–1.2	Secreted
Q8IXA5	SPACA3	Sperm acrosome membrane-associated protein 3	–2.6	–2.1	Cell-surface
Q5VZ72	IZUMO3	Izumo sperm–egg fusion protein 3	–2.6	–1.2	Cell-surface
P38567	SPAM1	Hyaluronidase PH-20	–2.6	–3.3	Secreted
Q8NEB7	ACRBP	Acrosin-binding protein	–2.6	–2.9	Secreted
Q9UKF5	ADAM29	Disintegrin and metalloproteinase domain-containing protein 29	–1.3	–1.2	Cell-surface
Q9H4B8	DPEP3	Dipeptidase 3	–0.2	0.4	Cell-surface

Interestingly, the top down-regulated protein in rs35033974*^hh^* spermatozoa was LY6K (14-fold change; *t* test *p* value = 0.03). LY6K is a GPI-anchored protein localized at the cell surface of testicular germ cells, with a similar expression pattern to TEX101 based on immunohistochemistry data available at the Human Protein Atlas. It was previously demonstrated in mice that LY6K disappeared from the germ cell surface in the absence of TEX101 protein in *Tex101* knockout mice (9, 36). Finally, we evaluated LY6K, ADAM29, and TEX101 proteins by Western blotting. Levels of these proteins were undetectable in rs35033974*^hh^* spermatozoa, while expression of DPEP3 monomers (75 kDa) and dimmers (150 kDa) was found at normal levels (Fig. 4*B*).

##### TEX101 and LY6K Localization in Human Spermatozoa

**Fig. 5.**
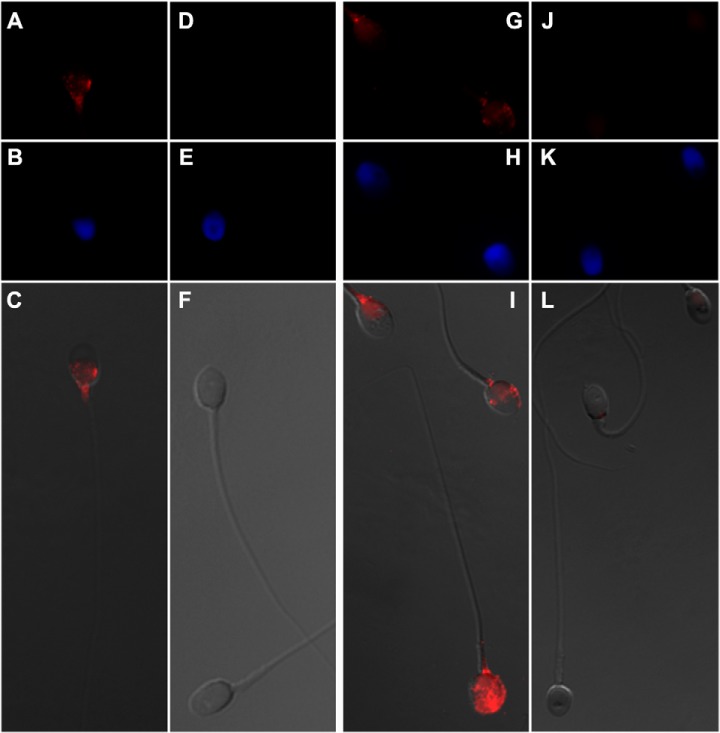
**Localization of TEX101 and LY6K proteins in human spermatozoa.** Immunofluorescence images were obtained with a 100× oil immersion objective. TEX101 (*A*) or LY6K (*G*) were labeled with primary antibodies and visualized with secondary antibodies conjugated to Alexa Fluor 594 (red). No primary antibodies were used for the negative controls (*D* and *J*). Spermatozoa nucleus (blue) was visualized with DAPI (*B*, *E*, *H*, and *K*). Panels *C*, *F*, *I*, and *L* represent merged brightfield and Alexa Fluor 594 fluorescence images. TEX101 and LY6K were localized in the neck, postequatorial and equatorial regions of spermatozoa. In some spermatozoa, LY6K was also localized in the acrosomal region. In the equatorial and postequatorial regions TEX101 and LY6K were localized to the plasma membrane.

Localization of TEX101 and LY6K proteins in human spermatozoa was visualized with a high-magnification immunofluorescence microscopy using a 100× oil immersion objective (Fig. 5). Because human spermatozoa had very high autofluorescence at the green channel (∼520 nm), red channel with Alexa Fluor 594 (peak emission at 617 nm) provided the most sensitive analysis of medium-abundance spermatozoa proteins, such as TEX101 and LY6K. As a result, the highest intensities of TEX101 and LY6K proteins were found in the neck region of spermatozoa, as well as postequatorial and equatorial regions. In some spermatozoa, LY6K was also occasionally localized in the acrosomal region. In the equatorial and postequatorial regions TEX101 and LY6K were localized to the plasma membrane.

## DISCUSSION

To identify genes essential for male fertility, ∼500 knockout mouse models have been investigated to date. Some models resulted in male infertility with severe defects in spermatogenesis, spermiogenesis and sperm maturation, or infertility with no apparent defects in motility, morphology, and sperm count (*Ace, Adam2, and Adam3*) (43, 44). Other models revealed subfertile phenotypes with reduced acrosome reaction and capacitation (*Acr*, *Pcsk4*), decreased motility (*Smcp*), delayed cumulus-oocyte complex dispersion (*Spam1*), weak zona pellucida binding (*Press21*), and reduced sperm-oocyte fusion (*Crisp1*) (45). Surprisingly, knockout studies in mice also revealed numerous sperm cell surface proteins not essential for fertilization *in vivo*, thus suggesting compensatory mechanisms and multifactorial nature of infertility (45).

Recently, the Human Protein Atlas profiled testis-specific genes and proteins and identified 1,079 genes with more than fivefold higher mRNA levels in testis as compared with all other human tissues (1). Due to their exclusive expression in testis, these proteins may be essential for spermatogenesis, remodeling of sperm surface proteome, sperm transit, and sperm-oocyte fusion (46). However, lack of cell lines expressing testis-specific proteins was the major bottleneck to study the molecular function of human-testis-specific proteins *in vitro*. Likewise, primary human germ cells isolated from orchiectomy samples could not be maintained in long-term cultures and studied with gene knockout or knockdown approaches.

Identification of natural “human knockouts” with homozygous loss-of-function mutations provided an alternative approach to study functional and pathological roles of human proteins (47). The most valuable mutations for such studies included protein truncating variants due to stop gain or frameshift mutations or single amino acid variants leading to the loss of activity or to protein misfolding followed by proteasomal degradation. In this work, we hypothesized that spermatozoa of men with natural knockouts or functional knockdowns of testis-specific genes could emerge as valuable models to study functional and pathological roles of human-testis-specific proteins.

Recent population-scale studies of genetic variation discovered numerous protein truncating variants or single amino acid variants in humans, provided their accurate frequencies in different ethnic groups and predicted their functional impact (22, 48, 49). The impact of such variants on expression and activity of germ-cell-specific proteins could be verified experimentally in spermatozoa or testicular tissues using mass spectrometry (50–52).

In this study, we focused on a germ-cell-specific protein TEX101 that we previously identified and validated as a seminal plasma biomarker for the differential diagnosis of azoospermia and male infertility (53–55). TEX101 was previously shown to be essential for the production of fertilization-competent spermatozoa through maturation of ADAM 3–6 proteins in mice (9, 10, 56–58). We have also recently completed a co-immunoprecipitation-mass spectrometry study on TEX101 and identified its physical interactome, including DPEP3 protein (59). Our motivation for the present study was to apply an orthogonal proteomic approach to discover TEX101-associated proteins, some of which could be proteins with weak and transient interactions and thus missed by co-immunoprecipitation. Here, we identified rs35033974 variant and discovered substantially lower levels of the G99V variant form of TEX101 protein in spermatozoa of heterozygous and homozygous men. We then hypothesized that rs35033974*^hh^* spermatozoa could be used as a knockdown model to identify proteins which co-degraded together with G99V TEX101 in rs35033974*^hh^* spermatozoa.

As a result, we identified and verified 7 TEX101-associated proteins that were significantly down-regulated in rs35033974*^hh^* spermatozoa. In agreement to previous studies in mice, we identified in our previous study (59) and in the present study two types of TEX101-interacting/associated proteins: (i) DPEP3 and alike proteins with strong physical interactions but no degradation in the absence of TEX101 and (ii) LY6K and alike proteins not found in the physical interactome but degraded in the absence of TEX101. It should be emphasized that identification of LY6K protein as a top candidate suggested the robustness of our experimental protocol. Indeed, previous studies in mice revealed that LY6K disappeared from the testicular germ-cell-surface in *Tex101* knockout mice (36).

Similar to TEX101, LY6K is GPI-anchored cell-surface protein expressed by testicular germ cells and is partially shed into seminal plasma during sperm maturation (36). Interaction of TEX101 with LY6K in mice was shown to be crucial for proper trafficking and posttranslational processing of LY6K. *Tex101*^−/−^ or *Ly6k*^−/−^ mice were infertile due to compromised migration of sperm in the oviduct (9, 36). TEX101-LY6K complex facilitated proper processing of ADAM3 protein. Interestingly, levels of TEX101 and LY6K proteins on the surface of spermatozoa but not levels of intracellular mRNA transcripts were mutually dependent. Thus, LY6K protein quickly degraded in *Tex101*^−/−^ mice, and vice versa (42). Another cell-surface GPI-anchored protein, a testis-specific dipeptidase DPEP3, formed a physical complex with TEX101 (58), but DPEP3 levels were not affected in *Tex101*^−/−^ mice (42). Thus, our data on human TEX101, LY6K and DPEP3 in WT and rs35033974*^hh^* spermatozoa (Fig. 4) confirmed previous observations in mice.

Global proteomic profiling of spermatozoa from four WT and four rs35033974*^hh^* men identified eight testis-specific ADAM proteins with adhesion (ADAM2, ADAM18, ADAM29, ADAM32) and metalloprotease (ADAM20, ADAM21, ADAM28, ADAM30) activities (35). Interestingly, it was only ADAM29 levels that decreased in rs35033974*^hh^* men, as discovered by global proteomic profiling (2.5-fold, *p* = 0.003) and verified by SRM and Western blotting. Because molecular function of human ADAM29 protein has never been previously reported, we suggest that ADAM29 protein should be further investigated as one of the potential functional orthologs of mouse ADAM 3–6 proteins.

There may be several possibilities to explain the discrepancy between molecular and clinical data on TEX101 protein: (i) Unlike mouse TEX101, human TEX101 protein and TEX101-LY6K interaction may not be essential for sperm maturation, ADAM processing and fertilization; (ii) low levels of TEX101 protein in rs35033974*^hh^* men may be compensated by alternative cell-surface chaperons; and (iii) rs35033974 is deleterious for protein structure; however, unlike mouse *Tex101*, human *TEX101* could be a nonessential gene (60, 61). Future studies should investigate if this highly frequent variant (1.6% homozygous genotype frequency in European population) predisposes males to infertility and becomes pathogenic in combination with other factors, for example, lowered sperm concentration in semen. Such multifactorial nature of male infertility has previously been discovered for germ-cell-specific proteins (62).

It should be noted that our study had the following limitations: (i) Even though label-free quantification using MaxQuant algorithm is recognized as an accurate proteome-wide quantification approach (15), its variability may still be relatively high, so all candidates should be verified by orthogonal assays, such as SRM or Western blotting; and (ii) global proteomic quantification of a very large number of proteins (8,046) and FDR-based cut-offs could result in numerous false-positive (for example, intracellular non-testis-specific proteins) and false-negative candidates (ADAM29 could be such a false-negative candidate).

To conclude, we presented the first human study to investigate the possible functional role of TEX101 protein as a cell-surface chaperone and identified degradation of LY6K and additional six germ-cell-specific proteins in rs35033974*^hh^* men. Spermatozoa of rs35033974*^hh^* men may be used as a unique model to elucidate further details on the role of human TEX101. Because TEX101 seminal plasma levels were found significantly lower in heterozygous than in WT men, rs35033974 status could be considered in TEX101 diagnostics. Our deep proteome of spermatozoa could be used as a resource to update currently missing testis-expressed proteins. Finally, our work may serve as a concept for future studies on functional effects of natural knockouts or knockdowns in humans. The presented approach may facilitate verification of the essential and nonessential testis-specific genes and proteins, which will advance biology of human reproduction.

## DATA AVAILABILITY

Raw mass spectrometry shotgun data and Proteome Discoverer and MaxQuant output files were deposited to the ProteomeXchange Consortium via PRIDE (www.ebi.ac.uk/pride/archive/login) with the dataset identifier PXD008333. PRM and SRM raw data were deposited to the Peptide Atlas with the dataset identifier PASS01112 (www.peptideatlas.org/PASS/PASS01112). Alternative link is ftp://PASS01112:NI5437g@ftp.peptideatlas.org. Processed Skyline files can be downloaded at Panorama Public (https://panoramaweb.org/3jbthK.url).

## Supplementary Material

supplemental Table S1

## Footnotes

^1^ The abbreviations used are:

TEX101: testis-expressed protein 101
LY6K: lymphocyte antigen 6K
ACN: acetonitrile
ADAM29: A disintegrin and metalloproteinase domain-containing protein 29
AGC: automatic gain control
CADD: combined annotation dependent depletion
CFTR: cystic fibrosis transmembrane conductance regulator
CID: collision-induced dissociation
DPEP3: dipeptidase 3
BH-adjusted *t*-test: Benjamini–Hochberg-adjusted *t*-test
FDR: false discovery rate
GPI: glycosylphosphatidylinositol
LED: light-emitting diode
LFQ: label-free quantification
MWU: Mann Whitney unpaired *t*-test
NHS: N-hydroxysuccinimide
PCR: polymerase chain reaction
PRM: parallel reaction monitoring
SIFT: sorting intolerant from tolerant
SP: seminal plasma
SNV: Single nucleotide variation
SRM: Selected reaction monitoring
WT: wild-type.
