# Supplementary material for: Identification of TEX101-associated Proteins Through Proteomic Measurement of Human Spermatozoa Homozygous for the Missense Variant rs35033974
Source: Mol Cell Proteomics. 2018 Nov 14;18(2):338–51. doi: 10.1074/mcp.RA118.001170 (PMC6356071; doi:10.1074/mcp.RA118.001170)
Supplement: supplemental Table S1 [file RA118.001170_index.html]

Supplement to Identification of TEX101-associated proteins through proteomic measurement of human spermatozoa homozygous for the missense variant rs35033974 | Molecular & Cellular Proteomics

## Supplemental Data

- Supplemental Figures - Supplemental Figures
- Supplemental Tables - Supplemental Tables
