# Supplementary material for: Identification of TEX101-associated Proteins Through Proteomic Measurement of Human Spermatozoa Homozygous for the Missense Variant rs35033974
Source: Mol Cell Proteomics. 2018 Nov 14;18(2):338–51. doi: 10.1074/mcp.RA118.001170 (PMC6356071; doi:10.1074/mcp.RA118.001170)
Supplement: supplemental Table S1 [file 141583_0_supp_225358_ph6hvq.docx]

Supplemental Figures for

**Identification of TEX101-associated proteins through proteomic measurement of human spermatozoa homozygous for the missense variant rs35033974**

Christina Schiza^1,2^, Dimitrios Korbakis^1,3^, Keith Jarvi^2,4^, Eleftherios P. Diamandis^1,2,3,5*^ and Andrei P. Drabovich^1,2,5*^

^1^Department of Laboratory Medicine and Pathobiology, University of Toronto, Toronto, Canada;

^2^Department of Pathology and Laboratory Medicine, Mount Sinai Hospital, Toronto, Canada;

^3^Lunenfeld-Tanenbaum Research Institute, Mount Sinai Hospital, Toronto, Canada;

^4^Department of Surgery, Division of Urology, Mount Sinai Hospital, University of Toronto, Toronto, Canada

^5^Department of Clinical Biochemistry, University Health Network, Toronto, Canada;

**Supplemental Figures include:**

**Figure S1.** Conservation of TEX101 residues

**Figure S2.** Examples of families with rs35033974^hh^ homozygous fathers, as determined by the 1000 Genomes Project

**Figure S3.** In silico predicted structure of TEX101 protein

**Figure S4.** Performance of individual peptides of TEX101 protein

**Figure S5.** Immunofluorescence analysis of TEX101 protein in spermatozoa of one rs35033974*^hh^* and one WT man.

**Supplemental Figure S1.** Conservation of TEX101 residues. Alignment of TEX101 protein sequences from various mammals revealed that glycine at position 99 (in red box) is highly conserved, and is present in all species, except *Oryctolagus cuniculus* (rabbit) and *Bos taurus* (bovine).

**Supplemental Figure S2.** Examples of families with rs35033974*^hh^* homozygous fathers, as determined by the 1000 Genomes Project (<http://phase3browser.1000genomes.org/Homo_sapiens/Variation/Population?db=core;r=19:43920112-43921112;v=rs35033974;vdb=variation;vf=9870510#664_tablePanel>). CEU: Utah residents with Northern and Western European ancestry


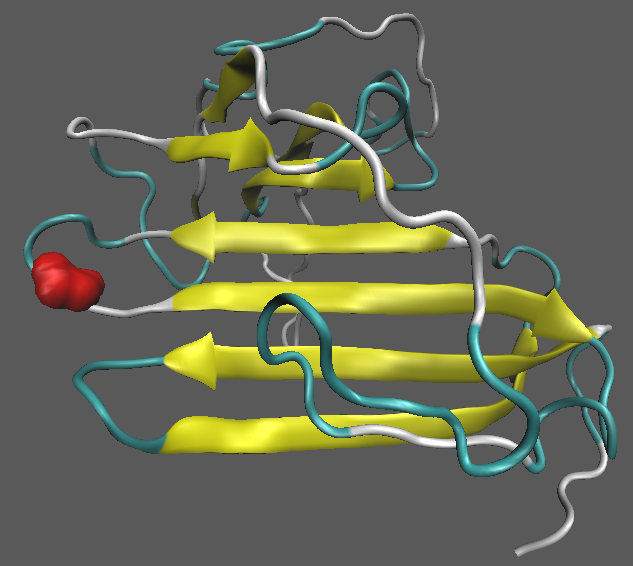


**Supplemental Figure S3.** *In silico* predicted structure of TEX101 protein (<https://swissmodel.expasy.org/repository/uniprot/Q9BY14>). Glycine-99 (*red*) is located within a PPGL beta-turn in the proximity of antiparallel beta sheet strands. Typically, a proline residue in beta-turns provides a rigid cyclic structure ideally suited for the turn, while small glycine residue lacking side chains flexibility of the turn. A large residue of valine at position 99 in the G99V variant form could introduce substantial steric constraints, eliminate the beta-turn and destabilize beta-sheets in the proximity of G99V.

**Supplemental Figure S4.** Performance of individual peptides of TEX101 protein, as measured by the global proteomic analysis in spermatozoa of four rs35033974^hh^ men (PI-401, PI-656, PI-702 and PI-714) and four WT men (PreV-012, -013, -030 and -064). Each sample was measured in technical duplicates.

**Supplemental Figure S5.** Immunofluorescence analysis of TEX101 protein in spermatozoa of one rs35033974*^hh^* man (PI-702) (**A-C**) and one WT man (PreV-012) (**D-F**). Images were obtained with a 60× oil immersion objective. Anti-TEX101 mouse monoclonal antibody 34ED229 was used as a primary antibody, while goat-anti-mouse secondary antibody labeled with Alexa-594 (red) was a secondary antibody. DAPI was used for nuclear staining (blue) in C, F and I. Negative control (G-I) included WT spermatozoa (PreV-012) labeled only with the secondary antibody. A, D and G represent merged brightfield and 594 nm images.
